# Fragment-Hopping-Based Discovery of a Novel Chemical Series of Proto-Oncogene PIM-1 Kinase Inhibitors

**DOI:** 10.1371/journal.pone.0045964

**Published:** 2012-10-24

**Authors:** Gustavo Saluste, Maria I. Albarran, Rosa M. Alvarez, Obdulia Rabal, Miguel Angel Ortega, Carmen Blanco, Guido Kurz, Antonio Salgado, Paolo Pevarello, James R. Bischoff, Joaquin Pastor, Julen Oyarzabal

**Affiliations:** Experimental Therapeutics Program, Spanish National Cancer Research Centre (CNIO), C/Melchor Fernández Almagro 3, Madrid, Spain; Spanish National Cancer Center, Spain

## Abstract

A new chemical series, triazolo[4,5-b]pyridines, has been identified as an inhibitor of PIM-1 by a chemotype hopping strategy based on a chemically feasible fragment database. In this case, structure-based virtual screening and *in silico* chemogenomics provide added value to the previously reported strategy of prioritizing among proposed novel scaffolds. Pairwise comparison between compound **3,** recently discontinued from Phase I clinical trials, and molecule **8,** bearing the selected novel scaffold, shows that the primary activities are similar (IC_50_ in the 20 to 150 nM range). At the same time, some ADME properties (for example, an increase of more than 45% in metabolic stability in human liver microsomes) and the off-target selectivity (for example, an increase of more than 2 log units in IC_50_ *vs.* FLT3) are improved, and the intellectual property (IP) position is enhanced. The discovery of a reliable starting point that fulfills critical criteria for a plausible medicinal chemistry project is demonstrated in this prospective study.

## Introduction

PIM-1, a cytoplasmic, highly conserved serine/threonine kinase, was first described as a retroviral insertion site for Moloney Murine Leukemia Virus (MoMuLV) that accelerated virus-induced T-cell lymphomagenesis, leading to its name **P**rovirus **I**ntegration site for **M**olony leukemia virus 1 [1]. Subsequently, it was noted that PIM-1 transgenic mice develop T-cell lymphoblastic lymphomas [2] and that PIM-1 cooperated with both N-MYC or c-MYC in murine leukemia virus-induced tumors [3], thus establishing PIM-1 as a proto-oncogene. The PIM-1 gene encodes a serine/threonine protein kinase [4], [5], [6]. PIM-1 has two closely related family members, PIM-2 and PIM-3. PIM-1 and PIM-2 share 61% of amino acid identity in their respective catalytic domains, whereas PIM-3 is 77% and 66% identical in the catalytic domain to PIM-1 and PIM-2, respectively. Mice in which the three PIM kinase genes have been knocked out are viable and fertile. Indeed, the strongest phenotype in triple knockout mice is a reduction in body size supporting a role for the PIM kinases in growth. Hematopoietic cells from triple knockout mice have an impaired response to certain growth factors *in vitro*, a fact that has been primarily attributed to the loss of PIM-1 [7].

PIM-1 is overexpressed in various hematopoietic malignancies, such as multiple myeloma, mantle cell lymphoma and diffuse large B-cell lymphomas, as well as in FLT 3/ITD positive acute myeloid leukemia (AML) [8], [9]. In mantle cell lymphoma, PIM-1 and Ki67 expression were predictive of poorer outcomes in a Phase II clinical trial of aggressive chemotherapy and rituximab [10]. There are also reports of overexpression of PIM-1 in solid tumors, such as in the prostate, pancreas and colon [9], [11]. Recently, it has been proposed that PIM-1 overexpression in the gastric glands of patients with gastric cancer is prognostic for lymph node metastasis and survival [12]. In prostate cancer, the expression of PIM-1 transcripts is related to the grade of prostate carcinomas [13]; higher PIM-1 expression is associated with higher WHO grades and higher Gleason scores, while upregulation of PIM-1 is related to the progression to a more aggressive form of prostate carcinoma. It has also been shown that the co-expression of PIM-1 and c-MYC is associated with higher Gleason scores [14].

In addition, a variety of studies have demonstrated that PIM-1 can act as a survival factor [5], [15]. This activity is thought to be mainly due to the phosphorylation of the pro-apoptotic protein Bad on Ser112 by Pim 1 [16], [17]. This phosphorylation facilitates the interaction between Bad and the 14-3-3 proteins leading to the proteosomal degradation of Bad and enhanced survival [18]. Recently, it has been shown that Pim 1 can phosphorylate the apoptosis signaling kinase Ask1 that results in protection from H_2_O_2_-induced cell death [19], indicating that PIM-1 may also be involved in protecting cells from apoptosis caused by stress-activated pathways.

In addition to its roles in proliferation and survival, PIM-1 has recently been found to have a role in CXCR12- and CXCR4-mediated homing and migration of FLT3/ITD-expressing AML cells [20]. This finding and other data suggest that PIM-1 could be involved in the migration of leukemic cells via regulation of the expression of CXCR4.

**Figure 1:**
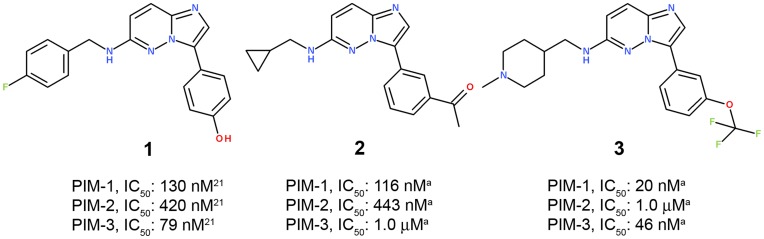
Compounds 1 and 2 are well-known, previously reported PIM-1 inhibitors. Compound 3 has been recently discontinued from Phase I clinical trials. These molecules bear an identical central core: imidazopyridazine. *^a^*IC_50_ values were obtained as described in the Methods section.

Due to its overexpression in various types of tumors, as well as its role in the regulation of pathways considered as relevant in cancer, inhibitors of PIM-1 are of interest as potential therapeutic agents [21]. Given this promising target profile, we began a drug discovery program targeting PIM-1. To identify inhibitors of PIM-1, 3,000 compounds were screened for their ability to inhibit the catalytic activity of PIM-1. These compounds were selected from the BioFocus collection, which is focused on kinases (Cambrigde, UK). The screen identified imidazopyridazine **1**, ETP-39010, as a potent biochemical inhibitor of PIM-1, IC_50_ of 130 nM [21]. However, an additional molecule in this chemical series, compound **2** (K00135) [22], [23], had previously been described as an inhibitor of PIM kinases (Figure 1). Several research groups (e.g. CNIO, Structural Genomics Consortium and SuperGen) [21], [22], [24] purchased this chemical series from the same supplier, BioFocus; thus, same chemotype was utilized as starting point in different drug discovery programs. From an intellectual property (IP) perspective, this fact may involve some risks.

While this project was underway, a PIM inhibitor with imidazopyridazine as its central core, compound **3** (SGI-1776) [25], [26], entered Phase I clinical trials (Figure 1). These trials reported that PIM inhibitors also have significant activity towards certain receptor tyrosine kinases. Thus, it is difficult to understand the contribution of PIM kinase inhibition to the biological effects observed with these compounds or to predict implications (e.g., toxicity) coming from this off-target promiscuity [21]. In addition, SuperGen’s compound **3** has recently been discontinued from Phase I clinical trials (in November 2010). The press release by SuperGen indicated that the “detailed cardiac and pharmacokinetic data evaluation of **3** in this trial has failed to demonstrate a safe therapeutic window” [27].

**Figure 2:**
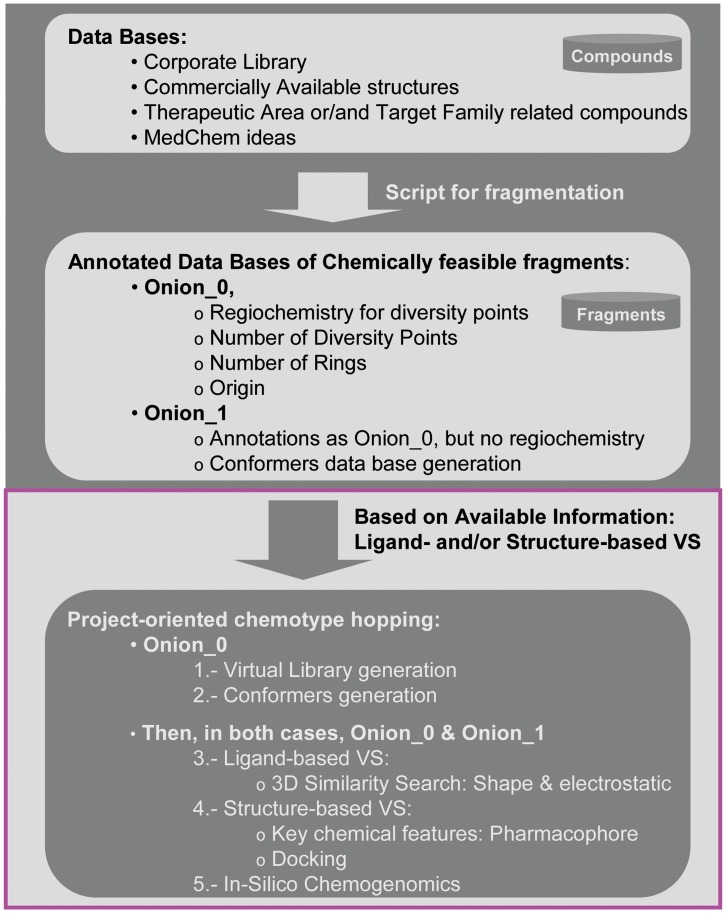
Flowchart of the proposed strategy with two main phases. 1. Generation of annotated DBs of chemically feasible fragments; 2. Based on previously generated DBs, ligand-based and structure-based VS strategies are applied together with an *in silico* chemogenomics approach to prioritize among the proposed chemotypes. This last part of the flowchart (magenta box) corresponds to a sequential stepwise process.

Thus, the identification of a novel chemical series to circumvent the issues of off-target promiscuity, as well as of negative cardiac-related and pharmacokinetic data, that simultaneously maintains its primary activity and provides strong IP is a critical challenge. Addressing this challenge may lead to us achieving an optimal starting point to reach our final goal: establishing PIM-1 inhibitors as therapeutic agents. To achieve this objective, the chemotype hopping strategy based on chemically feasible fragments [28], described in Figure 2, was utilized. In this case, as structural information was available, structure-based virtual screening (VS) approaches were utilized together with ligand-based three-dimensional (3D) similarity analysis to refine the prioritization process among the proposed novel scaffolds. Finally, *in silico* chemogenomics profiling was used as an additional guideline to select among the proposed chemotypes, leading to virtual compounds with optimal estimated off-target selectivity.

Herein, we describe a prospective case study where the proposed fragment-hopping approach led to the discovery of a novel chemical series of PIM-1 inhibitors. Thus, based on the new series reported in this manuscript, the next step of the drug discovery process started: a medicinal chemistry project was launched to explore initial hits described below. Details about the corresponding hit explosion from the identified starting points have recently been published [29].

## Methods

### Preparation of Databases

All fragments included in these databases were extracted from previously synthesized compounds and thus, by definition, they are chemically feasible. Compounds were extracted from the CNIO corporate database, which includes a virtual library of external real compounds, therapeutic area (TA) databases, a target family (TF) database (in this case, kinases), a target family related ligand database and a database based on MedChem experience. Before any fragmentation was performed, rare elements and salts were removed. Structures were standardized through tautomer generation and the formation of their corresponding canonical representations. Duplicates were eliminated through the use of a customized Pipeline Pilot [30] protocol. Fragment abstraction was performed at different levels from the original compound databases by using a publically available program [28] coded in the scientific vector language (SVL) of the MOE software system [31]. Two fragmentation levels were utilized: Onion0 and Onion1. Each database was created in duplicate with fragments derived from each of the two levels. The Onion0 fragmentation level yielded structures coming from the closest fragmentation around the central scaffold, resulting in “naked” chemotypes decorated only with their corresponding growing vectors or anchor points. Onion1 fragmentation delivered a more elaborate structure with not only the information for the atom at a distance of one atom from the central core but also the information regarding the functionality of the atom [28]. Functionalities close to the central core are sometimes a driving force in ligand-receptor interactions, together with the main chemotype.

All fragments included in the databases had at least one ring and were annotated with the most relevant information, including the regiochemistry, the position for anchor points from which chemotypes can be functionalized, the number of diversity points, the number of fused rings, the molecular weight, the source, the phase (pre-clinical, Phase I, II or III) and some other *in silico* estimated properties.

### CNIO Corporate Database

**Figure 3:**
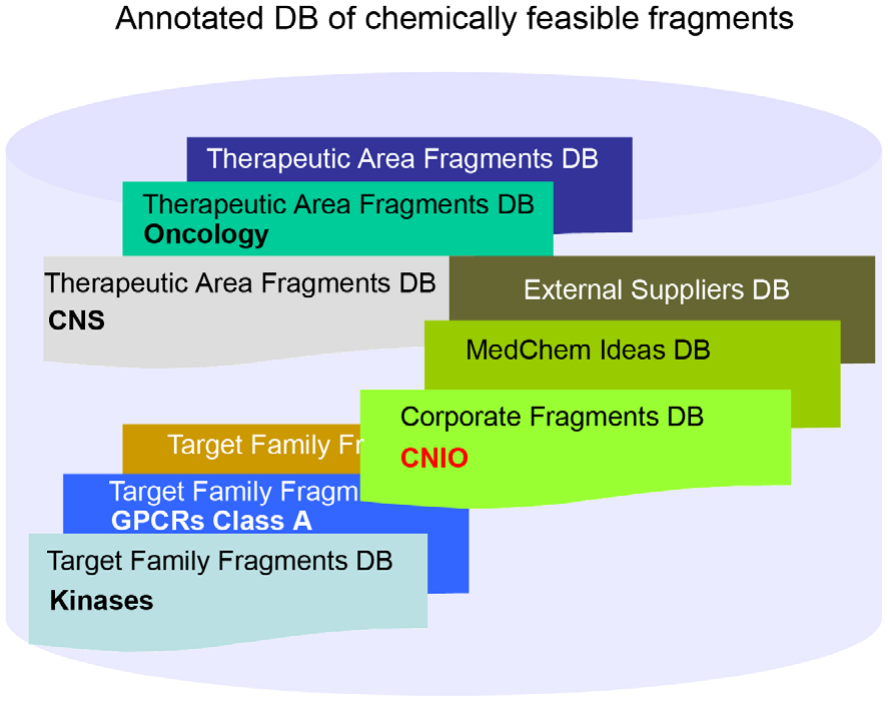
Annotated database of chemically feasible fragments.

Our library contains 42,168 unique compounds. In addition, our virtual CNIO library is composed of 10.8 million unique real compounds that are commercially available and/or reported (e.g., in PubMed, ChemBank, etc.). The combined libraries (in stock and virtual) comprise the CNIO corporate database. The program described above was utilized to obtain the corresponding CNIO Onion0 and Onion1 fragment databases. Data mining was required to archive only those useful fragments for chemotype hopping. The following criteria were applied: (i) chemical structures that bear at least one ring, (ii) that have a molecular weight between 60 and 300 and (iii) whose number of diversity points ranges from 1 to 4. The Onion0 CNIO corporate database contained 191,931 unique fragments fitting these criteria, and Onion1 was composed of 586,989 unique scaffolds. Finally, to define a target family related DB, in-house information from CNIO and the data from external databases, such as Kinase Knowledgebase (KKB) [32], were utilized to build the kinase-oriented fragment DB. Once the three fragment databases (corporate, TA- or TF-oriented and MedChem) were prepared (Figure 3), the project-oriented chemotype hopping began.

### Project-oriented Chemotype Hopping

Structural information for the target under study was available. Thus, the crystal structure corresponding to the halo-form for PIM-1 with compound **2** as the ligand [23] was utilized. The Protein Data Bank (PDB) code is 2C3I. In addition to the ligand-based virtual screening approach that relies on 3D similarity analyses, the final part of the flowchart described in Figure 2 (project-oriented chemotype hopping process, magenta box) takes into account structural information. Based on the structural data, different virtual screening approaches were utilized as part of this sequential stepwise process to assess key decision-making criteria for prioritizing the fragments.

According to the roadmap described in the magenta box (Figure 2), the first step of this process involved ligand-based virtual screening (3D similarity search based on shape and electrostatics). However, before utilizing this approach, different strategies were applied to the Onion0 and Onion1 fragment DBs. For the Onion0 fragment DB, the first step was defining which fragments from this database could be used in each case. Then, only those scaffolds meeting certain criteria were selected, e.g., those having a certain number of rings and number of diversity points. This decision was guided by the reference structure. The next step was the identification of the closest key minimal substitution pattern around the reference compound(s) at a distance of one atom from the central core. Then, this substitution pattern was utilized together with other potentially interesting linkers or substitutions to build a virtual library (VL) using the annotated anchor points and their regiochemistry from previously selected scaffolds. This process is illustrated below. For the Onion1 fragment DB, all anchor points were capped with methyl groups. Then, the corresponding capped Onion1 fragment DB was further utilized without additional manipulation [28].

**Figure 4:**
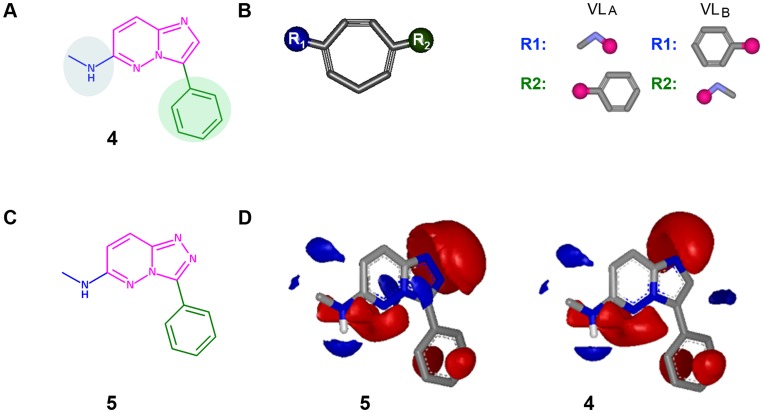
a) Key substructure 4, from reference compounds, was utilized as a template for the 3D similarity analysis (as illustrated in d); b) Representation of a potential scaffold from the Onion0 fragment DB. The Virtual Library was generated from the scaffolds generated from Onion0 as the sum of VL_A_ and VL_B_. For R-groups, the attachment points are shown as pink circles. c) Molecule **5** was ranked in the fourth position through the ligand-based VS; d) Electrostatic maps for compound **5** and reference substructure **4** were obtained with EON software [33]. Electrostatic grids are generated with two default contours: a positive one and a negative one. The positive contour is colored blue and the negative contour is colored red.

Compounds **1** and **2** were utilized as reference structures (Figure 1). These chemical structures have the same imidazopyridazine chemotype. Therefore, our reference substructure to perform scaffold-hopping was **4**; the key fragment to be replaced is shown in magenta (imidazopyridazine). Its critical substitution pattern was therefore part of reference substructure **4** (Figure 4A). Methylamine (in blue) and phenyl (in green) were kept constant in the reference compounds.

Those chemotypes that may have fit better with reference substructure–the fragments with one or two fused rings and two diversity points–were selected from the Onion0 database. In total, there were 32,340 unique chemotypes. These fragments were utilized to build virtual libraries bearing as decoration those linkers and key substitutions illustrated in reference substructure **4**, methylamine and phenyl, as they may have an impact on the electrostatic characteristics of the main chemotype of the reference structure. Therefore, two potential virtual libraries were constructed. All the scaffolds from the Onion0 fragment DB that met the previously reported criteria were used to generate the corresponding Virtual Libraries (VL): (a) VL_A_, keeping methylamine at position R_1_ and phenyl at R_2_ and (b) VL_B_, where phenyl will be born, in this case, at R_1_ and methylamine at position R_2_ (Figure 4B). Selection of R-groups to build these virtual libraries was based on the closest substitution pattern around the central cores from the reference compounds described in Figure 1; however, any R-group could be included in this analysis.

These libraries were constructed using MOE software [31]; their generation occurs very quickly and can be completed within several seconds. To remove any compound with unwanted chemistry (e.g., any nitrogen-oxygen bond) and any duplicates, a PipelinePilot [30] protocol was built and run, thereby obtaining the final, project-based, virtual library from annotated fragments. In total, this final virtual library contained 56,378 functionalized chemically feasible fragments.

Once the focused virtual library from the Onion0 fragment DB was built and the Onion1 DB was properly capped (586,989 structures), the corresponding conformers for each of these two sets of compounds were generated. Software developed by OpenEye [34], called Omega [35], was utilized to explore the conformational space around each functionalized chemotype within an energetic window of 25 Kcal/mol. Conformers with a root of mean squared deviation (rmsd) larger than 0.4 Å were stored until a maximum number of 500 was reached [36].

### 3D Similarity Analyses

Once all the conformers from the Onion0 project-oriented functionalized fragments and the Onion1 capped fragments were generated, we aligned them with the corresponding reference compound, **4**, before performing any further analysis. In this case, we utilized the ROCS (Rapid Overlay of Chemical Structures) software package [37] by OpenEye to perform the alignment, as this program is a fast and accurate [38] method for superimposing molecules. Shape similarity can be determined, in part, by comparing the shapes of those molecules. Once the overlap was optimized, the shape similarity was computed using the Tanimoto equation. However, ROCS does not contain an accurate notion of charge distribution and therefore is not a complete solution to the search for molecular similarity. The electrostatic fields of molecules can be calculated, and the similarity between the fields is expressed as the electrostatic Tanimoto. Electrostatics Tanimoto (ET) scores were calculated using the EON program [33] by OpenEye. Molecules were superimposed using ROCS, providing shape similarities in comparison to the reference compound. The electrostatic potentials were calculated using OpenEye’s ZAP Poisson-Boltzman solver implemented in EON. Taking into account the dampening of the electrostatic field by the aqueous solvent, the ET was calculated using an external dielectric of 80 and therefore may better represent the field experienced upon binding a protein [39]. A normalized shape Tanimoto of unity indicates that the molecules are identical, and the Tanimoto moves closer to zero as the molecules become less similar; electrostatic Tanimoto ranges from −0.3 to +1 indicate dissimilar and similar electrostatic fields [34]. From each original fragment library, top ranking compounds in terms of shape and electrostatics were selected.

Considering the information obtained in the previous report describing this fragment-hopping strategy [28], we were initially focused on those top-ranking compounds with scores for electrostatic Tanimoto (ET) higher than 0.62 and for shape higher than 0.75. Therefore, we examined the area with the highest values for 3D similarity to reference substructure **4**, to obtain the highest enrichment factors. Among the 643,367 structures from the Onion0 VL and the Onion1 fragment DB, only 63 structures met these requirements. The scores defining the ET and shape thresholds were defined for a different project, and the small number of top ranking structures meeting the requirements, scores were relaxed to include structures with an ET higher than 0.50 and a shape higher than 0.70 to decrease the number of potentially discarded hits (false negatives). Then, 198 chemical structures (top ranking 0.03%) met the 3D similarity requirements and progressed to the next step in the flowchart: structure-based VS.

The initial analysis of this 3D similarity search led to promising results, as a chemical series patented [40] and reported [41] by Vertex, substructure **5**, was ranked at the fourth position (Figures 4C and 4D). The ET was 0.734, and its corresponding TS was 0.964. This result was considered to be a blind validation for this case study, as this fragment had not been explicitly included.

### Structural Information

**Figure 5:**
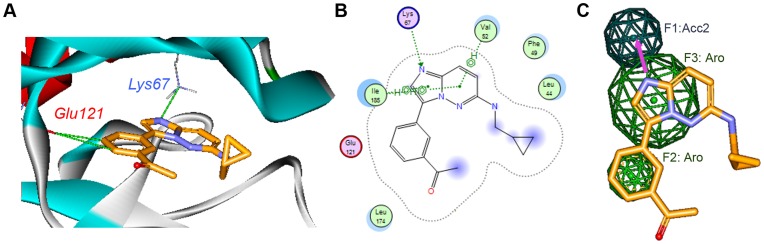
Structural Information. a) Imidazopyridazine ligand **2** bound to PIM-1 in the crystal structure, where explicit hydrogen bonds involved in the binding are highlighted; b) Bidimensional (2D) interaction map for the complex PIM-1**:**compound **2** extracted from 2C3I.pdb; c) 3-point pharmacophore deduced from the compound **2** bioactive conformation, using the CHD scheme, as implemented in MOE software (the hydrogen-bond acceptor is light-blue and aromatic ring pharmacophoric features are in green).

The crystal structure of PIM-1 determined in complex with compound **2** (2C3I.pdb) [23] was initially utilized for the structure-based virtual screening. The imidazopyridazine **2** binds to the ATP binding site of PIM1 and shows the typically turned in, inactive, Phe49 conformation which is incompatible with substrate binding. Compound **2** accepts a hydrogen bond from the side chain of Lys67 and, according to the distance between the heavy atoms (3.0 and 3.7 Å, respectively) and their nature, may donate a hydrogen bond to the backbone carbonyl of hinge residue Glu121. In fact, a bifurcated hydrogen bond forms in which two aromatic CH groups interact with a common main chain carbonyl [41]. In addition, there are a number of hydrophobic contacts, particularly with PIM-1 residues Leu44, Phe49, Val52, Ile104, Leu120 and Ile185. The unusual hinge architecture of PIM-1, which has a proline at the hinge position 123 allowing for formation of only a single hydrogen bond to ATP or other kinase inhibitors, and the unexpected binding mode of this inhibitor, where the key driving force for its binding pose may be its interaction with Lys67 (plausibly, a weaker interaction occurs with the hinge region), might be explored further to design specific inhibitors (Figures 5A and 5B).

Based on this information, three chemical features were identified as ligand requirements to bind to PIM-1. This information helped to define the corresponding pharmacophore: a hydrogen bond acceptor facing Lys67 together with two features accounting for aromatic rings, one of which is defined by a large sphere to fit not only bicycles but also aromatic monocycles and the other one is defined by the smallest sphere that accounts for the phenyl ring (Figure 5C). Having access to this structural data provides precise and critical information for the ligand: its bioactive conformation and the role of each chemical feature.

The charged-hydrophobic direction (CHD) scheme was utilized to directly build the 3-points pharmacophore from the conformation annotated in the PDB file. Before running the corresponding virtual screening, a specific conformer database was built for the 198 previously selected structures using Omega [35] with the above-described parameters. Once VS was performed, 66 compounds perfectly fit those three pharmacophoric features. Only these molecules were identified as hits.

Based on docking experiments, this reduced set of 66 molecules was prioritized according to their binding poses and scores. Before performing any docking, we first confirmed that the software, Gold 3.1 [42], was functioning for the PIM-1 complex. The binding site was defined using the available experimental information. The docking region used for PIM-1 was a 12-Å sphere around the carbon CB of Leu44 (atom 118). The GoldScore scoring function was used to rank docking poses without constraints to obtain an unbiased result and to explore all possible binding modes of the ligand, in this case, compound **2**. For each ligand, the top five best-docked structures out of 20 independent genetic algorithm runs were retrieved. For validation purposes, we compared the data for compound **2** docked to PIM-1 with the corresponding crystal structure [pdb entry 2C3I.pdb]. The RMSD between the reported compound **2** and the pose obtained, as shown by a unique consensus answer, was 1.70 Å. Thus, using the proposed set-up, this software was working properly for this PIM-1 complex. Once GOLD was validated as a docking tool for this complex, an identical set-up was utilized to perform the docking studies for the 66 selected compounds. For each ligand, the top five best-docked structures out of 20 independent genetic algorithm runs were retrieved. In this case, the consensus answer consisted of the top three ranked results out of five.

**Figure 6:**
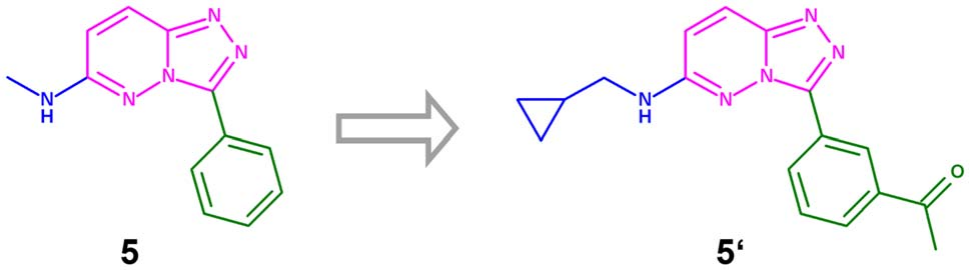
The 66 selected fragments, with their substructures shown in magenta, were functionalized according to the compound 2 substitution pattern before performing docking studies. The chemotype borne by compound 5 was functionalized accordingly and becomes 5′, after which it was docked.

Before docking experiments with the 66 chemical structures that fit all of the pharmacophoric requirements were run, the structures were properly functionalized according to the compound **2** substitution pattern. For example, the structure of compound **5** evolved to **5′** (Figure 6), and then the latter was docked. The same process was followed for all 66 selected molecules.

A detailed analysis of the results obtained from the docking studies showed that only 19 out of the 66 compounds yielded a consensus response for the proposed binding pose and maintained the critical interactions reported above (Figure 5). Finally, an in-house computational approach to polypharmacology, implemented as part of the compounds registration process [43], was applied to estimate off-target selectivity profiling for potential target compounds derived from the selected chemotypes. None of the proposed compounds had notable predicted promiscuity; consequently, all 19 scaffolds proceeded.

The final step in the process, which involved two different time-consuming analyses, therefore only focused on these 19 fragments. The last step involved the i) assessment of chemical feasibility based on the number of synthetic steps, critical reactions, etc. and their potential to “open” additional diversity points and ii) determining their IP position.

**Figure 7:**
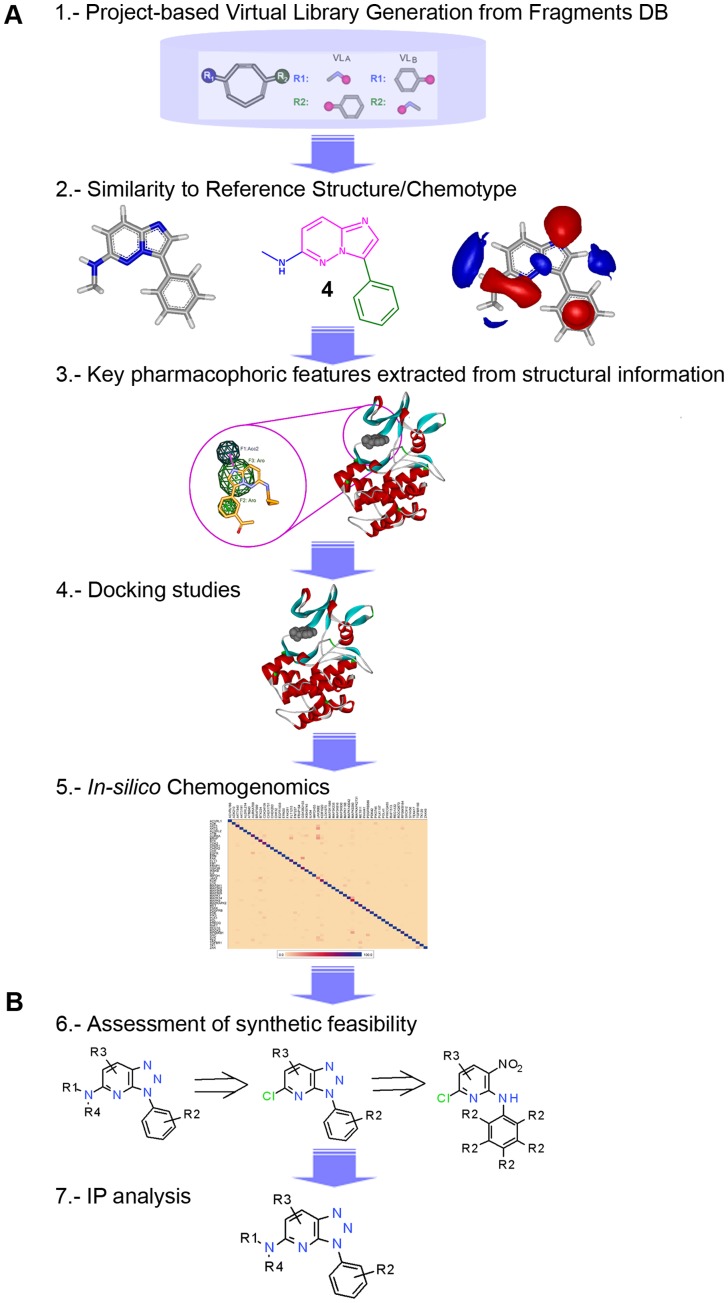
Roadmap used as a strategic guideline for project-oriented scaffold-hopping. This detailed overview describes the final part of Figure 2 (magenta box).

The roadmap shown in Figure 7 graphically represents the strategic guide utilized to perform this project-oriented scaffold prioritization, a sequential stepwise process split into two phases: a) a comprehensive *in silico* systematic strategy, in which a variety of virtual screening approaches were used to navigate a fragment DB that capitalized on knowledge from experienced medicinal chemists, in-house generated information, reported data, etc. and b) a time-consuming manual analysis.

## Results and Discussion

**Figure 8:**
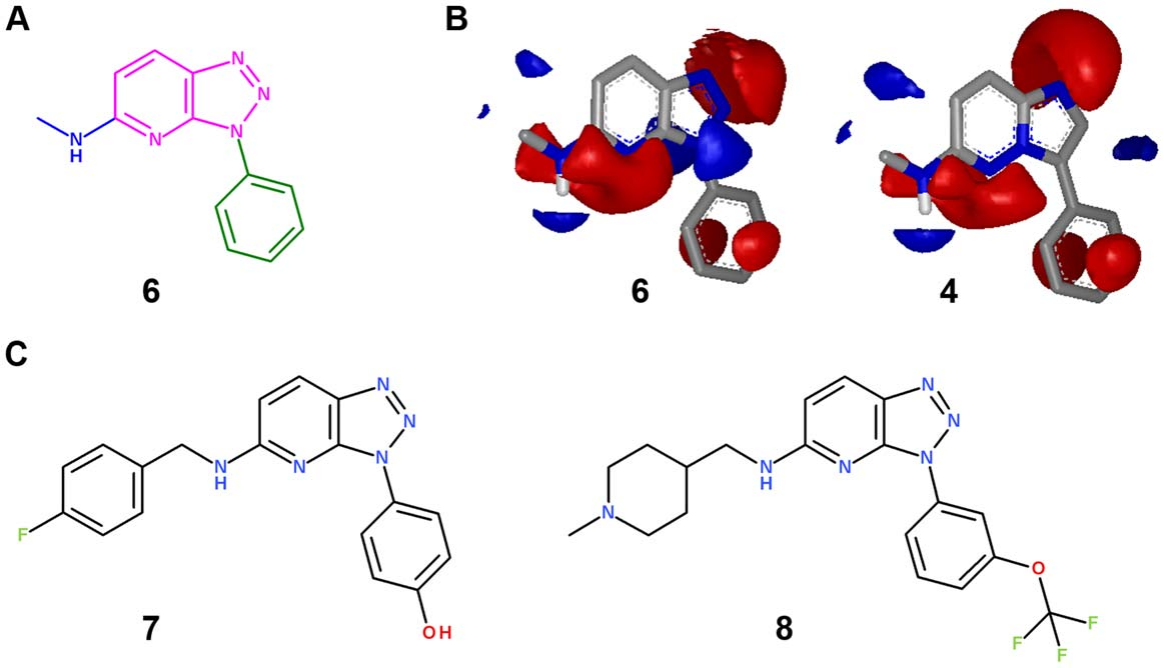
a) Molecule 6 bearing a triazolopyridine fragment as the central scaffold; b) Electrostatic maps for compound 6 and reference substructure 4 obtained with EON software; c) Proposed new chemical structures, bearing triazolopyridine, as PIM-1 inhibitors.

As a definitive outcome of this process, triazolopyridine was identified as a potential alternative scaffold to imidazopyridazines. In the first, ligand-based, virtual screening step, the corresponding molecule **6** (from the VL) bearing this fragment ranked 8^th^ in the 3D similarity search with an ET of 0.754 and a TS of 0.96 (Figure 8). This chemotype fulfilled all the critical criteria in the course of the stepwise process for project-oriented chemotype hopping, including ligand- to structure-based VS, synthetic feasibility and IP (Figure 7). Further studies involving this chemical series validated the *in silico* chemogenomics profiling (details below) and the structure-based VS approach as prospective tools. The docking studies fit perfectly with the experimental data (RMSD between the crystal structure and the corresponding docked molecule was 1.80 Å) maintaining all critical binding interactions described in Figure 5 [29].

To test if triazolopyridine is the alternative scaffold to imidazopyridazine, the synthesis of two key tool compounds bearing this fragment as the central core, together with well-known substitution patterns (transferring knowledge from compounds **1** and **3**), was evaluated. Therefore, compounds **7** and **8** were proposed (Figure 8) and synthesized (details in the materials and methods section).

Once compounds **7** and **8** were synthesized, a pairwise comparison was performed to strictly evaluate the real impact of this fragment-hopping where imidazopyridazine is replaced by triazolopyridine. The hit coming from our internal screening campaign, compound **1**, had been tested against a panel of 20 protein kinases at a concentration of 10 µM and showed poor off-target selectivity by inhibiting the three receptor tyrosine kinases FLT3, KIT and PDGFR-α, as well as the serine/threonine kinases DYRK1A and RPS6KA1, by more than 90% [21]. Compound **7** had the same substitution pattern as **1** and was assayed against the same panel of 20 kinases at the same concentration (Table 1).

**Table 1:** Compound 7, containing a triazolopyridine, is a selective PIM-1 inhibitor.

	Compound 1	Compound 7	
**PIM-1** (IC_50_, nM)	130	69	
**PIM-2** (IC_50_, nM)	420	333	
**PIM-3** (IC_50_, nM)	79	51	
**FLT-3** (IC_50_, nM)	1	1840	
**AKT1** a	0	0	
**ARK5** a	71	13	c
**B-RAF-V600E** a	54	8	b
**CK1-α1** a	33	8	b
**DYRK1A** a	99	73	b
**EGF-R** a	33	8	b
**FAK** a	48	38	
**FGF-R1** a	84	19	c
**IGF1-R** a	26	9	
**INS-R** a	67	32	b
**JAK2** a	48	30	
**JNK1** a	62	11	c
**KIT** a	95	51	b
**MET** a	32	21	
**MST1** a	82	29	c
**PAK1** a	7	5	
**PDGFR-α** a	98	53	b
**RPS6KA1** a	96	45	c
**SGK1** a	73	12	c

IC_50_ values were obtained as described in the Methods Section.

a The values reported are an average of two independent data points; those related to compound **1** have been previously reported.^21^

b This designation indicates that the improvement in percentage of inhibition was equal or greater than 25%.

c This designation indicates that the improvement in percentage of inhibition was equal or greater than 50%. Inhibitors were used at a final concentration of 10 µM. Details of the assay conditions can be found at www.ProQinase.com.

These results clearly highlight the impact and value of scaffold hopping. While the primary activity against PIM-1 and the rest of isoforms was kept constant, off-target selectivity was clearly improved. Due to the fact that compound **7** contained triazolopyridine instead of imidazipyridazine, it was more selective against all 20 kinases. In fact, 18 of them showed a percentage of inhibition lower than 55% (at 10 µM) and only one, DYRK1A, had an inhibition greater than 70%. Nevertheless, the percentage of inhibition against this target was reduced by more than 25% compared to compound **1**. The corresponding IC_50_s for FLT3 were determined, and the selectivity was improved by more than 3 log units. Experimental values for off-target selectivity were in agreement; overall accuracy was 81.8% with the estimated *in silico* chemogenomics profiling generated for compound **7** (details in Supplementary Information, Table S1).

As previously described, compound **3** (SGI-1776) [25], [26] entered Phase I clinical trials but was discontinued because “detailed cardiac and pharmacokinetic data evaluation of **3** in this trial has failed to demonstrate a safe therapeutic window” [27]. Therefore, to obtain more detailed information on this critical point, preliminary *in vitro* ADME profiling was performed for molecule **3**. This profiling was mainly focused on hERG activity and on pharmacokinetics parameters, such as metabolic stability and PAMPA (an *in vitro* model for passive diffusion). Once compound **8** was synthesized, a pairwise comparison was performed to evaluate the impact of the central core replacement on the *in vitro* ADME (Table 2) data and on off-target selectivity (Table 3).

**Table 2:** Compound 3 vs. Compound 8: primary activity and *in vitro* ADME profiling.

	PIM-1^a^	PIM-2^a^	PIM-3^a^	Met. Stab.^b^ *(Human)*	Met. Stab.^b^ *(Mouse)*	Met. Stab.^b^ *(Rat)*	hERG^c^	PAMPA^d^
Compound **3**	20	1000	46	31	5	23	2.6	0.0015
Compound **8**	155	4950	724	77	27	35	5.4	0.74

a IC_50_ values (nM) were obtained as described in the Methods section.

b Metabolic stability is reported as percentage of the original compound, at 1 µM, that remained after the incubation with liver microsomes from different species for 15 minutes.

c IC_50_ values are reported as µM and were calculated in duplicate from radioligand binding assays using [^3^H]-astemizole.

d Pe values, reported as 10^-6 ^cm/s were calculated by LC/MS/MS after 4 hours of incubation at 2 µM. Details for these three *in vitro* ADME assay conditions can be found at http://www.wuxiapptec.com/.

**Table 3:** Compound 8, containing a triazolopyridine, showed better selectivity profiling than compound 3.

	Compound 3	Compound 8	
**PIM-1** (IC_50_, nM)	20	155	
**PIM-2** (IC_50_, nM)	1000	4950	
**PIM-3** (IC_50_, nM)	46	724	
**FLT-3** (IC_50_, nM)	5	1320	
**AKT1**	0^a^	0^b^	
**ARK5**	42^a^	18^b^	
**B-RAF-V600E**	41^a^	10^b^	
**CK1-α1**	0^a^	8^b^	
**DYRK1A**	19^a^	3^b^	
**EGF-R**	32^a^	18^b^	
**FAK**	68^a^	34^b^	
**FGF-R1**	65^a^	16^b^	
**IGF1-R**	53^a^	25^b^	
**IKK-β**	0^a^	3^b^	
**JAK2**	3^a^	0^b^	
**KIT**	96^a^	44^b^	c
**MEK1**	11^a^	33^b^	
**MET**	12^a^	6^b^	
**MST1**	60^a^	20^b^	
**PAK1**	8^b^	2^b^	
**PDGFR-α**	92^a^	20^b^	c
**RPS6KA1**	48^a^	37^b^	
**SGK1**	55^a^	0^b^	c

IC_50_ values were obtained as described in the Methods Section. The values reported are an average of two independent data points. Inhibitors were used at a final concentration of 10 µM^a^ (3) and 5 µM^b^ (8), respectively. ^c^This designation indicates that the improvement in percentage of inhibition was greater than 50%. IC_50_ values were obtained as described in the Methods Section. Details of assay conditions can be found at www.ProQinase.com.

The most noteworthy data came from the apparent instability of compound **3** in human liver microsomes. After 15 minutes of incubation, only 31% of the original compound remained, and the results were worse for the other species. However, by replacing imidazopyridazine by triazolopyridine, these data were largely improved. When compound **8** was assayed against human liver microsomes, 77% of the original compound **8** remained after 15 minutes of incubation (Table 2). The hERG affinity was almost identical (slightly improved, decreased) when the scaffold replacement was performed. The IC_50_ for compound **8** was 5.4 µM, whereas it was 2.6 µM for compound **3**. In addition, the PAMPA data showed a better passive diffusion when the assayed compound contained the triazolopyridine fragment, from an effective permeability (Pe) lower than 0.1 (10^−6^ cm/s) to a Pe close to 1 (Pe values greater than one are considered optimal). Primary activity against PIM-1 was slightly worse for compound **8** than for compound **3**.

Off-target selectivity profiling was also performed for compounds **3** and **8** against a panel of 20 kinases (Table 3). Compound **3** was assayed at a final concentration of 10 µM while compound **8** was assayed at a final concentration of 5 µM, which was 2 times lower. When taking the differences in screening concentrations into consideration, we can only make unequivocal conclusions about FLT3 (based on the IC_50_ determination). Against this target, compound **8** was more selective than molecule **3** by more than 2 log units. In addition, there were three cases (KIT, PDFGR-α and SGK1) where the increment in percentage of inhibition, and therefore also in selectivity, was larger than 50%, which may suggest some improvement in selectivity for compound **8** versus compound **3** against those three additional targets. The slight improvement observed for the remainder of the screened targets was likely due to the difference in concentrations. These data confirm the trends reported above in Table 1. Thus, based on two direct pairwise comparisons on scaffolds containing identical substitution patterns, the data indicate that triazolopyridine provides a better off-target selectivity profile than does imidazopyridazine. Experimental values for off-target selectivity are in agreement; overall accuracy is 83.3% for the estimated *in silico* chemogenomics profiling generated for compound **8** (details in Supplementary Information, Table S2).

### Conclusion

Application of this fragment-hopping strategy provides an excellent platform to be routinely utilized in drug discovery projects. Where structural information was publically available, the stepwise process for the last part of the strategy described in Figure 2 was based on a comprehensive *in silico* approach, in which not only ligand-based virtual screening and computational approach to polypharmacology but also structure-based approaches were utilized to prioritize among proposed novel scaffolds.

Through this prospective analysis of an actual case study, the impact that this fragment-hopping strategy had in a drug-discovery program is exemplified. In looking for novel PIM-1 inhibitors, this strategy led us to a) compounds from a new chemically feasible series, where the primary activity was kept equipotent, b) a chemotype with good IP position (in fact, a new patent has been filled on it) [44], c) improvements in off-target selectivity and d) positive changes in pharmacokinetic parameters, mainly focused on *in vitro* metabolic stability in liver microsomes. Thus, this case perfectly fits with the definition of scaffold hopping: generating an alternative chemical structure, ideally with optimal IP position, while preserving the original profile as a ligand and the binding affinity against the primary target, as well as improving its drug-like properties, not only in *in vitro* ADME but also in off-target selectivity. Being pragmatic, obviousness and/or similarity independent, the final goal was achieved: a novel chemical series meeting the critical criteria to launch a medicinal chemistry project has been prospectively discovered.

Consequently, further exploration of this chemical series was performed following these initial hits. A detailed structure-relationship analysis (SAR) was obtained during the hit to lead evaluation process and has recently been published [29]. Additional work exploring other selected scaffolds will be reported in due course.

## Materials and Methods

### Chemistry

#### General procedure

**Figure 9:**
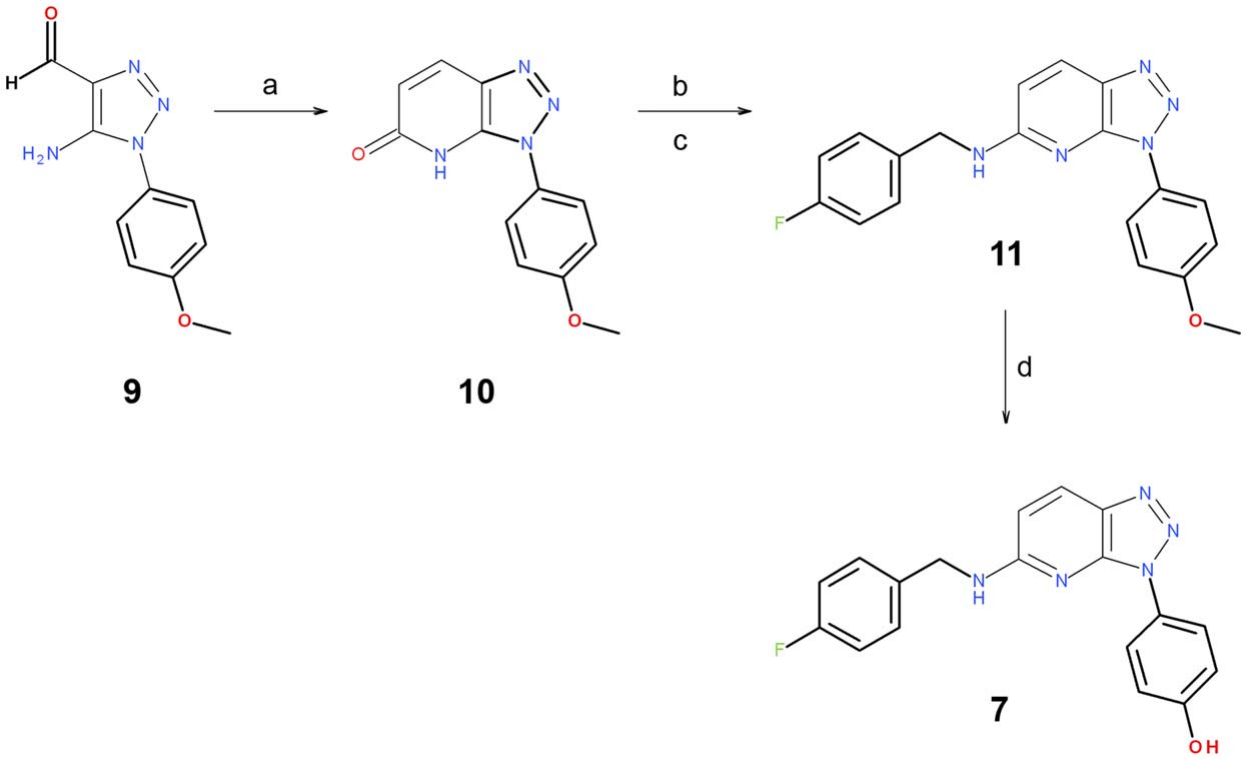
Reagents and conditions. (a) Triethyl Phosphonoacetate, NaEtO and EtOH; (b) TfOH and pyridine; (c) primary amine and dioxane; (d) BBr_3_ and DCM.

**Figure 10:**
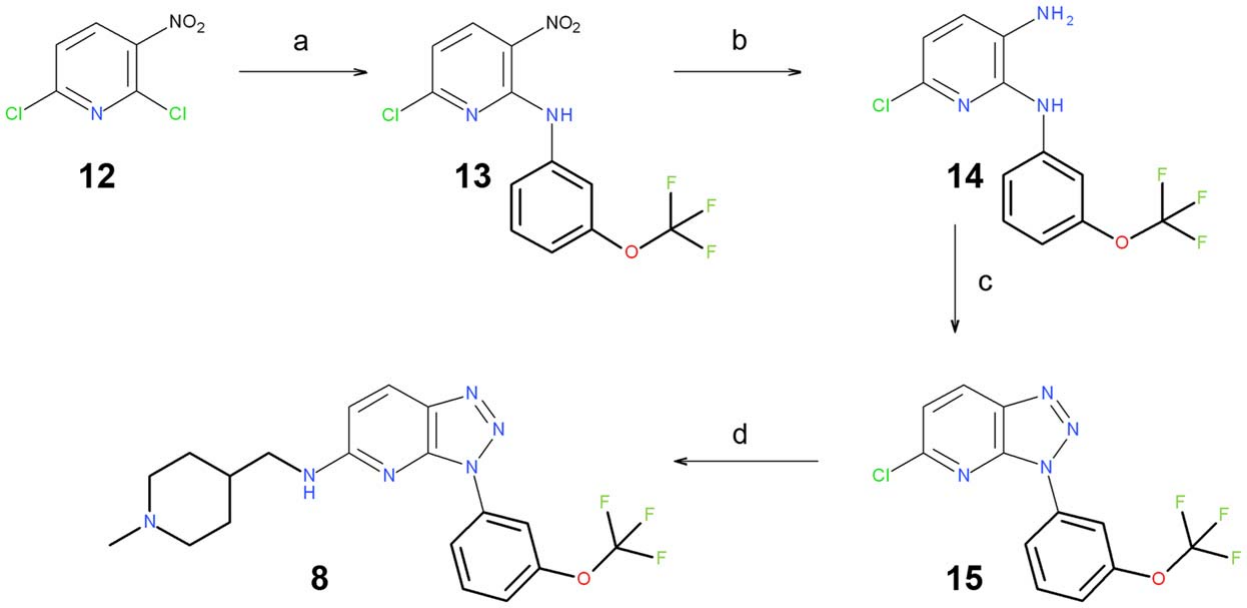
Reagents and conditions. (a) Aromatic amine, NaHCO_3_ and EtOH; (b) H_2_, Ni-Raney and EtOAc; (c) NaNO_2_, AcOH and H_2_O; (d) primary amine, TEA and EtOH.

Microwave-assisted reactions were performed in a single-mode reactor: Initiator™ Sixty microwave reactor (Biotage). A description of the instrument can be found at www.biotage.com. Thin layer chromatography (TLC) was carried out on silica gel 60 F_254_ plates (Merck) using reagent grade solvents. Flash column chromatography was performed on silica gel, particle size 60 Å, mesh 230–400 (Merck), using standard techniques. Automated flash column chromatography was performed using ready-to-connect cartridges from Varian, on irregular silica gel, particle size 15–40 µm (normal phase disposable flash columns), on a Biotage SPX flash purification system. ^1^H NMR spectra were recorded on a Bruker AVANCE II 300 or AVANCE 700 II spectrometer with standard pulse sequences, operating at 300 MHz and 700 MHz, respectively. Chemical shifts (δ) are reported in parts per million (ppm) downfield from tetramethylsilane (TMS), which was used as internal standard. HPLC analysis was performed using an Agilent HP 1100 system comprising a binary pump with a degasser, an autosampler, a column oven, a diode array detector (DAD) and a column, as specified in the respective methods below. Flow from the column was split to a MS spectrometer. The MS detector (Agilent 6120 Quadropole) was configured with an electrospray source or API/APCI. Nitrogen was used as the nebulizer gas. The source temperature was maintained at 150°C. Data acquisition was accomplished with ChemStation LC/MSD quad software. Purity for the assayed target compounds **1**, **3**, **7**, and **8** was >95%, as described in the Supporting Information (Quality Control S1). References compounds **1** and **3** were synthesized in house following previously reported methods [21], [24]. Compounds **7** and **8** were synthesized according to the strategies depicted in Figures 9 and 10.

The key step for the preparation of target compound **7** was the formation of the carbon-carbon bond for positions 4 and 5 of the bicycle. Then, the subsequent cyclization to obtain the central core was properly functionalized. This process was achieved with a Horner-Wadsworth-Emmons reaction using commercially available 5-Amino-1-(4-methoxy-phenyl)-1H-[1], [2], [3]triazole-4-carbaldehyde **9** with triethyl phosphonoacetate. Subsequently, triazolopyridone **10** was obtained through an intramolecular cyclization. Further treatment of this triazolopyridone with triflic anhydride and its subsequent coupling with a primary amine yielded triazolopyridine **11,** whose methoxy group was cleaved by a Lewis acid to achieve target molecule **7** (Figure 9).

An alternative synthetic strategy was proposed to achieve target compound **8** (Figure 10). Amination of the commercially available 2,6-dichloro-3-nitropyridine **12**, followed by the reduction of the NO_2_ group of **13,** yielded diamine **14**. The treatment of **14** with sodium nitrite [45] yielded triazole **15**. Finally, a second amination yielded the desired molecule **8**.

#### 3-(4-Methoxy-phenyl)-3,4-dihydro-[1], [2], [3]triazolo[4,5-b]pyridin-5-one (10)

5-Amino-1-(4-methoxy-phenyl)-1H-[1], [2], [3]triazole-4-carbaldehyde **9** (1.00 g; 4.580 mmol), purchased from GalChimia (www.galchimia.com), was suspended in dry EtOH (80 mL). Triethyl phosphonoacetate (1.33 g; 5.96 mmol) was added, followed by sodium ethoxide (4.3 mL; 11.46 mmol; 21% by wt). The mixture was heated at 80°C overnight. The solvent was evaporated and the residue was taken up in DCM and water. HCl (0.5N) was added to adjust the pH to 4. The product **10** was isolated by vacuum filtration and 0.91 g was obtained, (82%). ^1^H NMR (300 MHz, dmso-d_6_): δ 3.85 (s, 3H), 6.81 (d, *J* = 8.9 Hz, 1H), 7.19 (d, *J* = 8.8 Hz, 2H), 7.94 (d, *J* = 8.8 Hz, 2H), 8.38 (d, *J* = 8.9 Hz, 1H).

#### 4-Fluoro-benzyl)-[3-(4-methoxy-phenyl)-3H-[1], [2], [3]triazolo[4,5-b]pyridin-5-yl]-amine (11)

Triflic anhydride (1.16 g; 4.13 mmol) was added to dry pyridine (7 mL) at 0°C, and the mixture was stirred for 10 min. 3-(4-Methoxy-phenyl)-3,4-dihydro-[1], [2], [3]triazolo[4,5-b]pyridin-5-one (0.50 g; 2.06 mmol) was added, and the ice bath was removed. After 2 h, the solvent was evaporated and the oily residue was taken up in DCM and HCl (0.1 N). Extraction with DCM, drying and evaporation yielded the corresponding triflate in quantitative yield. The triflate (0.100 g; 0.27 mmol) was weighed into a reaction vial, and dioxane (3 mL) was added, followed by 4-fluorobenzylamine (0.134 g; 1.07 mmol). The reaction was stirred at 55°C overnight. The solvent was evaporated and the crude mixture was partitioned between DCM and saturated NaHCO_3_. Drying and evaporation of the organic layer, followed by purification (Biotage 12S, 10% EtOAc in hexane 5 CV, then ramp to 100% EtOAc over 10 CV) afforded 40 mg of the product **11**, (42%). MS *m/z* 350.1 [M + H]^+^.

#### 4-[5-(4-Fluoro-benzylamino)-[1], [2], [3]triazolo[4,5-b]pyridin-3-yl]-phenol (7)

(4-Fluoro-benzyl)-[3-(4-methoxy-phenyl)-3H-[1], [2], [3]triazolo[4,5-b]pyridin-5-yl]-amine (0.040 g; 0.11 mmol) was dissolved in dry DCM (1.5 mL) and boron tribromide solution (0.60 mL; 0.60 mmol; 1 M in DCM) was added. The reaction was stirred at room temperature for 4 days. MeOH (1 mL) was added and the mixture was stirred for 1 h before evaporating the solvent. The residue was partitioned between DCM and saturated NaHCO_3_ to yield a yellow solid. Purification was achieved on silica (EtOAc:Cyhexane 7∶3), and 14 mg of compound **7** (37%) was obtained. ^1^H NMR (300 MHz, Acetone-d_6_): δ 4.67 (d, *J* = 5.6 Hz, 2H), 6.78 (d, *J* = 9.1 Hz, 1H), 7.02–7.11 (m, 4H), 7.40–7.50 (m, 3H), 7.97–8.02 (m, 3H). MS *m/z* 336.1 [M + H]^+^.

#### (6-Chloro-3-nitro-pyridin-2-yl)-(3-trifluoromethoxy-phenyl)-amine (13)

2,6-Dichloro-3-nitropyridine (2.0 g; 10.36 mmol), 3-(trifluoromethoxy)aniline (1.87 g; 10.57 mmol) and NaHCO_3_ (0.87 g; 10.36 mmol) were added to dry EtOH (70 mL). The resulting mixture was stirred for three weeks at room temperature. The solvent was evaporated and the residue was washed with cold ethanol and water to afford 2.45 g of compound **13** (71%), as an intensely yellow solid (2.45 g; 71%). ^1^H NMR (300 MHz CDCl_3_): δ 6.86 (d, *J* = 8.6 Hz, 1H), 6.99–7.07 (m, 1H), 7.39 (t, *J* = 8.1 Hz, 1H), 7.46–7.50 (m, 1H), 7.76 (s, 1H), 8.47 (d, *J* = 8.6 Hz, 1H), 10.28 (s, 1H).

#### 6-Chloro-N*2*-(3-trifluoromethoxy-phenyl)-pyridine-2,3-diamine (14)

(6-Chloro-3-nitro-pyridin-2-yl)-(3-trifluoromethoxy-phenyl)-amine (1.20 g; 3.597 mmol), compound **13**, was dissolved in EtOAc (900 mL) and EtOH (150 mL). Catalytic hydrogenation was carried out on an H-Cube continuous flow hydrogenator using a Ni-Raney cartridge at 50 bar H_2_-pressure and 30°C, with a flow rate of 1 mL/min. The reaction was complete after three cycles. The solvent was evaporated, and the dark-green oily residue slowly crystallized to give 0.96 g of compound **14** (88%). MS *m/z* 304.1 [M + H]^+^.

#### 5-Chloro-3-(3-trifluoromethoxy-phenyl)-3H-[1], [2], [3]triazolo[4,5-b]pyridine (15)

6-Chloro-N*2*-(3-trifluoromethoxy-phenyl)-pyridine-2,3-diamine (0.660 g; 2.173 mmol) was dissolved in glacial AcOH (15 mL), and the solution was cooled in an ice bath. To this slurry, sodium nitrite (0.180 g; 2.608 mmol) was added as a solution in water (3 mL). The ice bath was removed, and the reaction continued for 2 h. Evaporation of the solvent yielded a residue that was purified on silica by applying a gradient ranging from 100% cyclohexane to 100% ethyl acetate. Evaporation yielded 0.62 g of compound **15** (91%). MS *m/z* 315. [M + H]^+^.

#### (1-Methyl-piperidin-4-ylmethyl)-[3-(3-trifluoromethoxy-phenyl)-3H-[1], [2], [3]triazolo[4,5-b]pyridin-5-yl]-amine (8)

5-Chloro-3-(3-trifluoromethoxy-phenyl)-3H-[1], [2], [3]triazolo[4,5-b]pyridine (0.050 g; 0.159 mmol) and (1-methyl-4-piperidinyl)methanamine (0.041 g; 0.318 mmol) were dissolved in dry EtOH (2 mL). Triethylamine (22 uL; 0.159 mmol) was added, and the reaction was heated in a 90°C sand bath for 72 h. The solvent was evaporated, and the crude product was treated with DCM/NaOH (0.1 M). Drying and evaporation resulted in a crude product that was purified on silica by eluting with DCM:MeOH (4∶1 with 1% triethylamine). Evaporation afforded 36.9 mg of compound **8** (57%). ^1^H NMR (300 MHz CDCl_3_): δ 1.23–1.45 (m, 2H), 1.68–1.80 (m, 2H), 1.89–1.99 (m, 3H), 2.25 (s, 3H), 2.87 (d, *J* = 11.6 Hz, 2H), 3.40 (t, *J* = 6.1 Hz, 2H), 5.17 (t, *J* = 5.6 Hz, 1H), 6.46 (d, *J* = 9.0 Hz, 1H), 7.19–7.23 (m, 1H), 7.54 (t, *J* = 8.3 Hz, 1H), 7.98 (d, *J* = 9.0 Hz, 1H), 8.27–8.30 (ddd, *J* = 8.3, 1.9, 0.9 Hz, 1H), 8.46 (s, 1 H). MS *m/z* 407.2 [M + H]^+^.

### Biochemical Assays

#### PIM

Kinase activity was measured with the commercial ADP Hunter™ Plus assay (DiscoveRx Ref. #33-016), a homogeneous assay measuring ADP accumulation, as a universal product of kinase activity. The assay was conducted following the manufacturer’s recommendations and adapting the protein and substrate concentrations for optimal conditions. The kinase assay buffer was 15 mM HEPES, pH 7.4, 20 mM NaCl, 1 mM EGTA, 0.02% Tween-20, 10 mM MgCl2 and 0.1 mg/ml BGG (bovine γ-globulin). All PIM kinase assays were performed with 100 µM PIMtide (ARKRRRHPSGPPTA) as the peptide substrate and 100 µM ATP. The protein concentrations were 50, 350 and 500 ng/µl for PIM-1, 2 and 3, respectively. To calculate the IC_50_ of the ETP-compounds, serial 1∶5 dilutions were prepared and the reaction was started with the addition of ATP. The reaction was incubated for 1 h at 25°C. Reagents A and B (DiscoveRx) were sequentially added to the wells, and the plates were incubated for 30 min at 37°C. Fluorescence counts were read in a Victor instrument (Perkin Elmer) with the recommended settings (544 and 580 nm as the excitation and emission wavelengths, respectively). Values were plotted against the inhibitor concentrations and fit to a sigmoid dose–response curve using GraphPad software [46].

#### FLT3

Kinase activity was measured with the commercial ADP Hunter™ Plus assay (DiscoveRx Ref. #33-016), a homogeneous assay measuring ADP accumulation, as a universal product of kinase activity. The assay was conducted following the manufacturer’s recommendations and adapting the protein and substrate concentrations for optimal conditions. The kinase assay buffer was 15 mM HEPES, pH 7.4, 20 mM NaCl, 1 mM EGTA, 0.02% Tween-20, 10 mM MgCl2 and 0.1 mg/ml BGG (bovine γ-globulin). All PIM kinase assays were performed with 100 µM ABLtide (EAIYAAPFAKKK) from GenScript as the peptide substrate and 100 µM ATP. Protein concentration (from Invitrogen, PV3182) was 0.4 ng/µl. To calculate the IC_50_ of the ETP-compounds, serial 1∶3 dilutions were prepared and the reaction was started with the addition of ATP. The reaction was incubated for 1 h at 37°C. Reagents A and B (DiscoveRx) were sequentially added to the wells, and the plates were incubated for 30 min at 25°C. Fluorescence counts were read in an EnVision 2104 Multilabel Reader (Perkin Elmer) with the recommended settings (544 and 580 nm as the excitation and emission wavelengths, respectively). Values were plotted against the inhibitor concentrations and fit to a sigmoid dose–response curve using ActivityBase software [47].

## Supporting Information

Table S1 ^a^IC_50_ values were obtained as described in Experimental Section; ^b^Percentages of inhibition as the mean of two independent experiments (details of assay conditions can be found at www.ProQinase.com). ^c^Application scope, from a biological space point of view, for this “in silico chemogenomics” model [43] is defined by 90 kinases. ^d^In this case, only 11 overlap with the assayed panel described; thus, estimations could not be determined (ND) for some targets. ^e^This designation indicates that predictive model did not properly classify the compound 7 *vs* the corresponding target. ^f^This designation indicates that predictive model properly classified compound 7 *vs* the corresponding target; where hit criteria is >50% inhibition (ligand at a fixed concentration of 10 µM). In this case, estimations fail in two cases, out of 11; then, overall accuracy is: 81.8%.
(DOC)

Table S2 ^a^IC_50_ values were obtained as described in Experimental Section; ^b^Percentages of inhibition as the mean of two independent experiments (details of assay conditions can be found at www.ProQinase.com). ^c^Application scope, from a biological space point of view, for this “in silico chemogenomics” model [43] is defined by 90 kinases. ^d^In this case, only 12 overlap with the assayed panel described; thus, estimations could not be determined (ND) for some targets. ^e^This designation indicates that predictive model did not properly classify the compound 8 *vs* the corresponding target. ^f^This designation indicates that predictive model properly classified compound 8 *vs* the corresponding target; where hit criteria is >40% inhibition (ligand at a fixed concentration of 5 µM). In this case, estimations fail in two cases, out of 12; then, overall accuracy is: 83.3%.
(DOC)

Quality Control S1 **HPLC/MS conditions, purity and retention times for reported synthetic intermediates 10, 11, 13, 14 and 15, as well as HPLC traces for the assayed target compounds 1, 3, 7, and 8, are included.**
(DOC)
