# Supplementary material for: Fragment-Hopping-Based Discovery of a Novel Chemical Series of Proto-Oncogene PIM-1 Kinase Inhibitors
Source: PLoS One. 2012 Oct 24;7(10):e45964. doi: 10.1371/journal.pone.0045964 (PMC3480357; doi:10.1371/journal.pone.0045964)
Supplement: Table S1 — aIC50 values were obtained as described in Experimental Section; bPercentages of inhibition as the mean of two independent experiments (details of assay conditions can be found at www.ProQinase.com). cApplication scope, from a biological space point of view, for this “in silico chemogenomics” model [43] is defined by 90 kinases. dIn this case, only 11 overlap with the assayed panel described; thus, estimations could not be determined (ND) for some targets. eThis designation indicates that predictive model did not properly classify the compound 7 vs the corresponding target. fThis designation indicates that predictive model properly classified compound 7 vs the corresponding target; where hit criteria is >50% inhibition (ligand at a fixed concentration of 10 µM). In this case, estimations fail in two cases, out of 11; then, overall accuracy is: 81.8%. (DOC) [file pone.0045964.s001.doc]

**Table S1**

|  |  | **Compound 7** |  |  |
| --- | --- | --- | --- | --- |
| **Targets** | **IC50 (nM)^a^** | **% Inbibition @ 10μM^b^** | **In-Silico Chemogenomics^c^** |  |
| FLT-3 | 1840 |  | no hit | e |
| AKT1 |  | 0 | no hit | f |
| ARK5 |  | 13 | ND^d^ |  |
| B-RAF-V6000E |  | 8 | no hit | f |
| CK1-Alpha1 |  | 8 | ND^d^ |  |
| DYRK1A |  | 73 | ND^d^ |  |
| EGF-R |  | 8 | no hit | f |
| FAK |  | 38 | no hit | f |
| FGFR1 |  | 19 | no hit | f |
| IGF1-R |  | 9 | no hit | f |
| INS-R |  | 32 | ND^d^ |  |
| JAK2 |  | 30 | no hit | f |
| JNK1 |  | 11 | no hit | f |
| KIT |  | 51 | no hit | e |
| MET |  | 21 | no hit | f |
| MST1 |  | 29 | ND^d^ |  |
| PAK1 |  | 5 | ND^d^ |  |
| PDGFR-Alpha |  | 53 | ND^d^ |  |
| RPS6KA1 |  | 45 | ND |  |
| SGK1 |  | 12 | ND |  |
