# Supplementary material for: Fragment-Hopping-Based Discovery of a Novel Chemical Series of Proto-Oncogene PIM-1 Kinase Inhibitors
Source: PLoS One. 2012 Oct 24;7(10):e45964. doi: 10.1371/journal.pone.0045964 (PMC3480357; doi:10.1371/journal.pone.0045964)
Supplement: Table S2 — aIC50 values were obtained as described in Experimental Section; bPercentages of inhibition as the mean of two independent experiments (details of assay conditions can be found at www.ProQinase.com). cApplication scope, from a biological space point of view, for this “in silico chemogenomics” model [43] is defined by 90 kinases. dIn this case, only 12 overlap with the assayed panel described; thus, estimations could not be determined (ND) for some targets. eThis designation indicates that predictive model did not properly classify the compound 8 vs the corresponding target. fThis designation indicates that predictive model properly classified compound 8 vs the corresponding target; where hit criteria is >40% inhibition (ligand at a fixed concentration of 5 µM). In this case, estimations fail in two cases, out of 12; then, overall accuracy is: 83.3%. (DOC) [file pone.0045964.s002.doc]

**Table S2**

|  |  | **Compound 8** |  |  |
| --- | --- | --- | --- | --- |
| **Targets** | **IC50 (nM)^a^** | **% Inbibition @ 5μM^b^** | **In-Silico Chemogenomics^c^** |  |
| FLT-3 | 1320 |  | no hit | e |
| AKT1 |  | 0 | no hit | f |
| ARK5 |  | 18 | ND^d^ |  |
| B-RAF-V6000E |  | 10 | no hit | f |
| CK1-Alpha1 |  | 8 | ND^d^ |  |
| DYRK1A |  | 3 | ND^d^ |  |
| EGF-R |  | 18 | no hit | f |
| FAK |  | 34 | hit | e |
| FGFR1 |  | 16 | no hit | f |
| IGF1-R |  | 25 | no hit | f |
| IKK-Beta |  | 3 | no hit | f |
| JAK2 |  | 0 | no hit | f |
| KIT |  | 44 | hit | f |
| MEK1 |  | 33 | no hit | f |
| MET |  | 6 | no hit | f |
| MST1 |  | 20 | ND^d^ |  |
| PAK1 |  | 2 | ND^d^ |  |
| PDGFR-Alpha |  | 20 | ND^d^ |  |
| RPS6KA1 |  | 37 | ND^d^ |  |
| SGK1 |  | 0 | ND^d^ |  |
