# Supplementary material for: Fragment-Hopping-Based Discovery of a Novel Chemical Series of Proto-Oncogene PIM-1 Kinase Inhibitors
Source: PLoS One. 2012 Oct 24;7(10):e45964. doi: 10.1371/journal.pone.0045964 (PMC3480357; doi:10.1371/journal.pone.0045964)
Supplement: Quality Control S1 — HPLC/MS conditions, purity and retention times for reported synthetic intermediates 10, 11, 13, 14 and 15, as well as HPLC traces for the assayed target compounds 1, 3, 7, and 8, are included. (DOC) [file pone.0045964.s003.doc]

Fragment-Hopping-Based Discovery of a Novel Chemical Series of Proto-oncogene PIM-1 Kinase Inhibitors.

Gustavo Saluste, M. I. Albarran, Rosa M. Alvarez, Obdulia Rabal,^1^ Miguel Angel Ortega, Carmen Blanco, Guido Kurz,^2^ Antonio Salgado, Paolo Pevarello,^3^ James R. Bischoff,^4^ Joaquin Pastor and Julen Oyarzabal^1^,*

# Table of Contents

**S1**. HPLC/MS Conditions………………………….…………………………………………………………………………...S2

**S2.1**. Purity for reported synthetic intermediates: **10, 11, 13, 14** and **15** ....…………………..……..S3

**S2.2**. Purity for assayed target compounds **1**, **3, 7** and **8**. ……...………..…...……………………………..S3

**S3**. HPLC traces for key target compounds **1**, **3, 7** and **8**…….……………………...……………….………...S4

**S4.** In-Silico Chemogenomics: **Table S1** and **Table S2** …………………………………………………………….S6

S1

**S1. HPLC/MS Conditions**

## General procedure

HPLC-analysis was performed using an Agilent HP 1100 system comprising a binary pump with degasser, an autosampler, a column oven, a diode array detector (DAD) and a column specified in the respective methods below. Flow from the column was split to a MS spectrometer. The MS detector (Agilent 6120 Quadropole) was configured with an electrospray source or API/APCI. Nitrogen was used as the nebulizer gas. The source temperature was maintained at 150 ºC. Data acquisition was accomplished with ChemStation LC/MSD quad software.

## HPLC Method **1**

12 minutes LC/MS run

Reverse-phase HPLC was carried out on Gemini-NX C18 (100 x 2.0 mm; 5um). Solvent A: water with 0.1% formic acid; solvent B: acetonitrile with 0.1% formic acid. Gradient: at 50 ºC, 5% of B to 100% of B over 8 min at 0.6 mL/min; then 100% B at 0.7 mL/min over 2 min, DAD.

## HPLC Method **2**

6 minutes LC/MS run

Reverse-phase HPLC was carried out on Gemini-NX C18 (100 x 2.0 mm; 5um). Solvent A: water with 0.1% formic acid; solvent B: acetonitrile with 0.1% formic acid. Gradient: at 50 ºC, 10% of B to 95% of B over 4 min at 0.5 mL/min; then 100% B at 0.8 mL/min over 2 min, DAD.

## HPLC Method **3**

15 minutes LC/MS run

Reverse-phase HPLC was carried out on Gemini-RP C18 (150 x 4.6 mm; 5um). Solvent A: water with 0.1% formic acid; solvent B: acetonitrile with 0.1% formic acid. Gradient: at 50 ºC, 5% of B to 100% of B over 15 min at 1.0 mL/min. DAD.

## HPLC Method **4**

10 minutes LC/MS run

Reverse-phase HPLC was carried out on Gemini-NX C18 (100 x 2.0 mm; 5um). Solvent A: water with 0.1% formic acid; solvent B: acetonitrile with 0.1% formic acid. Gradient: at 50 ºC, 5% of B to 100% of B over 8 min at 0.8 mL/min; then 100% B at 0.9 mL/min over 2 min, DAD.

S2

**S2.1.** Purity for reported synthetic intermediates: **10, 11, 12, 13, 14** and **15**. MS, HPLC and ^1^H-NMR analysis.

| Comp. | HPLC-MS [M + H]^+^ | | HPLC Purity | HPLC Method | HPLC System | ^1^H-NMR |
| --- | --- | --- | --- | --- | --- | --- |
|  | Rt | [M + H]^+^ | UV DAD |  |  |  |
| **10** | N/A | N/A | N/A | N/A | N/A | OK |
| **11** | 2.58 | 350.1 | 95% | 1 | ESI^+^ | N/A |
| **13** | N/A | N/A | N/A | N/A | N/A | OK |
| **14** | 4.74 | 304.1 | 92 % | 2 | ESI^+^ | N/A |
| **15** | 4.92 | 315.1 | 94 % | 2 | ESI^+^ | N/A |

**S2.2.** Purity determination for assayed target compounds: **1**, **3, 7** and **8**. MS, HPLC and ^1^H-NMR analysis.

| Comp. | HPLC-MS [M + H]^+^ | | HPLC Purity | HPLC Method | HPLC System | ^1^H-NMR |
| --- | --- | --- | --- | --- | --- | --- |
|  | Rt | [M + H]^+^ | UV DAD |  |  |  |
| **1** | 7.05 | 335.2 | 100% | 3 | ESI^+^ | OK |
| **3** | 2.18; 2.35 | 406.2 | 95.31% | 1 | ESI^+^ | OK |
| **7** | 6.72 | 336.1 | 100% | 1 | ESI^+^ | OK |
| **8** | 3.52 | 407.2 | 95.79% | 4 | ESI^+^ | OK |

S3

**S.3.** HPLC traces for key target compounds **1**, **3, 7** and **8**.

Compound **1**


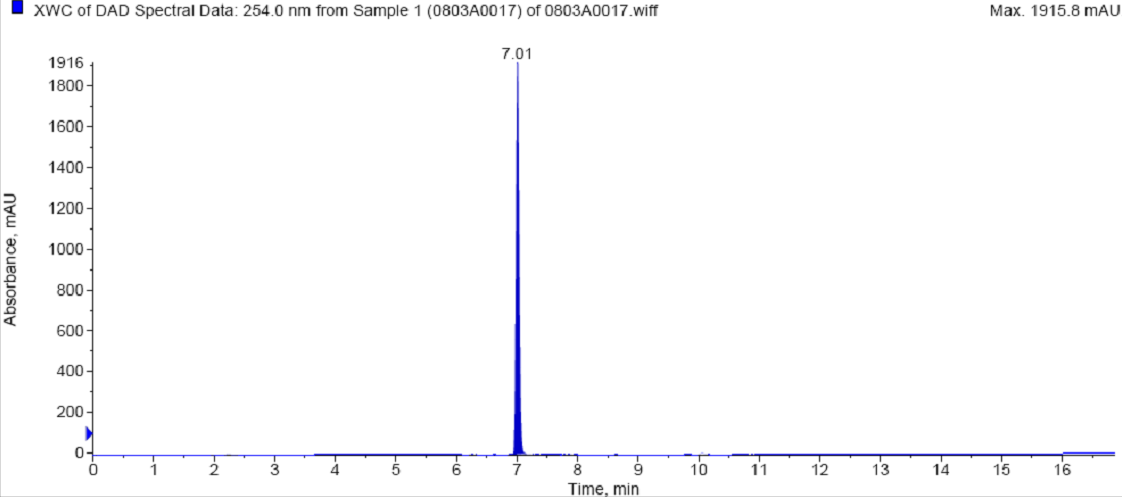


Compound **3**

***
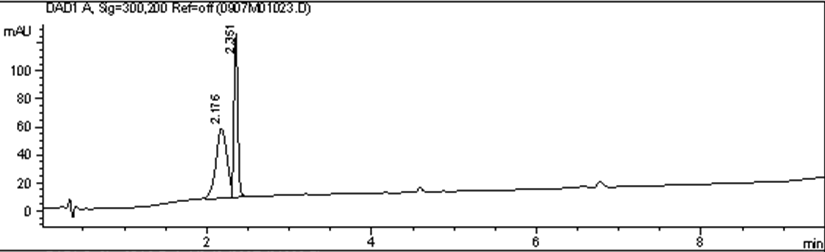
***

where, obtained fragmentation patterns for those two peaks are identical (image below).

In addition, NMR data is unequivocal and perfectly fits with reported information for this reference compound.


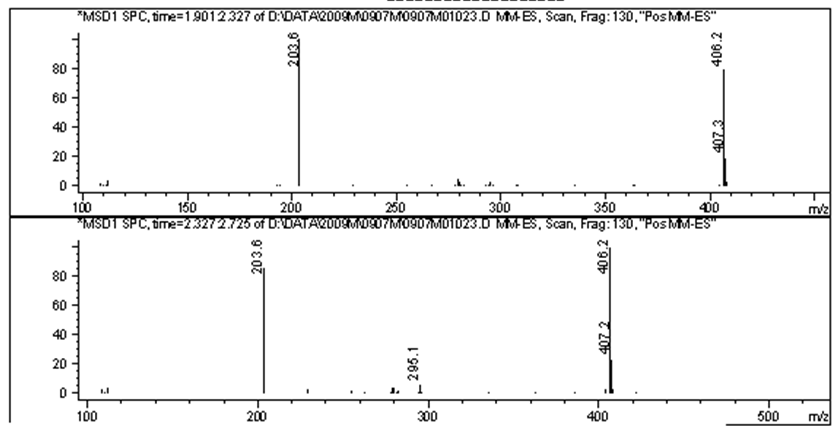


S4

Compound **7**


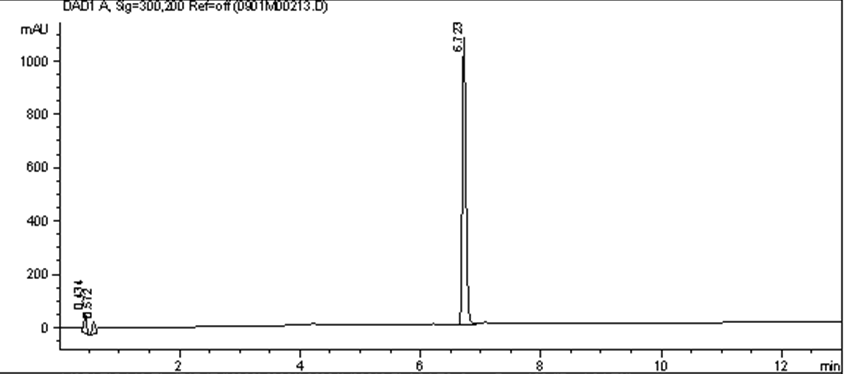


Compound **8**


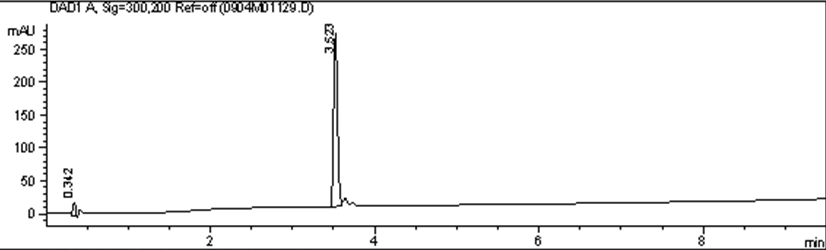


S5

**In-Silico Chemogenomics.**

**Table S1**

|  |  | **Compound 7** |  |  |
| --- | --- | --- | --- | --- |
| **Targets** | **IC50 (nM)^a^** | **% Inbibition @ 10μM^b^** | **In-Silico Chemogenomics^c^** |  |
| FLT-3 | 1840 |  | no hit | e |
| AKT1 |  | 0 | no hit | f |
| ARK5 |  | 13 | ND^d^ |  |
| B-RAF-V6000E |  | 8 | no hit | f |
| CK1-Alpha1 |  | 8 | ND^d^ |  |
| DYRK1A |  | 73 | ND^d^ |  |
| EGF-R |  | 8 | no hit | f |
| FAK |  | 38 | no hit | f |
| FGFR1 |  | 19 | no hit | f |
| IGF1-R |  | 9 | no hit | f |
| INS-R |  | 32 | ND^d^ |  |
| JAK2 |  | 30 | no hit | f |
| JNK1 |  | 11 | no hit | f |
| KIT |  | 51 | no hit | e |
| MET |  | 21 | no hit | f |
| MST1 |  | 29 | ND^d^ |  |
| PAK1 |  | 5 | ND^d^ |  |
| PDGFR-Alpha |  | 53 | ND^d^ |  |
| RPS6KA1 |  | 45 | ND |  |
| SGK1 |  | 12 | ND |  |

^a^IC_50_ values were obtained as described in Experimental Section; ^b^Percentages of inhibition as the mean of two independent experiments (details of assay conditions can be found at www.ProQinase.com). ^c^Application scope, from a biological space point of view, for this “in silico chemogenomics” model [43] is defined by 90 kinases. ^d^In this case, only 11 overlap with the assayed panel described; thus, estimations could not be determined (ND) for some targets. ^e^This designation indicates that predictive model did not properly classify the compound 7 *vs* the corresponding target. ^f^This designation indicates that predictive model properly classified compound 7 *vs* the corresponding target; where hit criteria is >50% inhibition (ligand at a fixed concentration of 10µM)

FLT3 is predicted as no-hit; however, although selectivity window is achieved *vs* compound **1** (more than 3 log units), according to hit definition (IC_50_ < 10µM) compound **7** is a FLT3 hit. In case of percentage of inhibition using ligand at a fixed concentration of 10µM; then, according to hit definition (% inhibition > 50) compound **7** is a KIT hit.

Therefore, in this case (compound **7**), estimations fail in two cases, out of 11. Then, overall accuracy is: 81.8%

S6

**Table S2**

|  |  | **Compound 8** |  |  |
| --- | --- | --- | --- | --- |
| **Targets** | **IC50 (nM)^a^** | **% Inbibition @ 5μM^b^** | **In-Silico Chemogenomics^c^** |  |
| FLT-3 | 1320 |  | no hit | e |
| AKT1 |  | 0 | no hit | f |
| ARK5 |  | 18 | ND^d^ |  |
| B-RAF-V6000E |  | 10 | no hit | f |
| CK1-Alpha1 |  | 8 | ND^d^ |  |
| DYRK1A |  | 3 | ND^d^ |  |
| EGF-R |  | 18 | no hit | f |
| FAK |  | 34 | hit | e |
| FGFR1 |  | 16 | no hit | f |
| IGF1-R |  | 25 | no hit | f |
| IKK-Beta |  | 3 | no hit | f |
| JAK2 |  | 0 | no hit | f |
| KIT |  | 44 | hit | f |
| MEK1 |  | 33 | no hit | f |
| MET |  | 6 | no hit | f |
| MST1 |  | 20 | ND^d^ |  |
| PAK1 |  | 2 | ND^d^ |  |
| PDGFR-Alpha |  | 20 | ND^d^ |  |
| RPS6KA1 |  | 37 | ND^d^ |  |
| SGK1 |  | 0 | ND^d^ |  |

^a^IC_50_ values were obtained as described in Experimental Section; ^b^Percentages of inhibition as the mean of two independent experiments (details of assay conditions can be found at www.ProQinase.com). ^c^Application scope, from a biological space point of view, for this “in silico chemogenomics” model [43] is defined by 90 kinases. ^d^In this case, only 12 overlap with the assayed panel described; thus, estimations could not be determined (ND) for some targets. ^e^This designation indicates that predictive model did not properly classify the compound 8 *vs* the corresponding target. ^f^This designation indicates that predictive model properly classified compound 8 *vs* the corresponding target; where hit criteria is >40% inhibition (ligand at a fixed concentration of 5µM)

FLT3 is predicted as no-hit; however, although selectivity window is achieved *vs* compound **3** (more than 2 log units), according to hit definition (IC_50_ < 10µM) compound **8** is a FLT3 hit. In case of percentage of inhibition using ligand at a fixed concentration of 5µM; then, according to hit definition (% inhibition > 40) compound **8** is a KIT hit and it s not a FAK hit.

Therefore, in this case (compound **8**), estimations fail in two cases, out of 12. Then, overall accuracy is: 83.3%

S7
